# Supplementary material for: Wide Fontanels, Delayed Speech Development and Hoarse Voice as Useful Signs in the Diagnosis of KBG Syndrome: A Clinical Description of 23 Cases with Pathogenic Variants Involving the ANKRD11 Gene or Submicroscopic Chromosomal Rearrangements of 16q24.3
Source: Genes (Basel). 2021 Aug 17;12(8):1257. doi: 10.3390/genes12081257 (PMC8394041; doi:10.3390/genes12081257)
Supplement: Supplementary file 1 [file genes-12-01257-s001.zip › Table S1 Summary of panels used and quality data for patients examined with next-generation sequencing.pdf]

Table S1. Summary of panels used and quality data for patients examined with next-generation sequencing. Samples that were processed with custom panels were sequenced at 2x75 cycles.

Patient	Identified Variant	Name of the Panel	Allele Depth/Overall Depth of Coverage for the Variant	Instrument	Mean Coverage	% of Sequences Covered At Least 20x
1	c.1903_1907del (p.Lys635Glnfs*26)	SS_CRANIO_V2	190/414	NextSeq550	340x	99.8%
2	c.1903_1907del (p.Lys635Glnfs*26)	CRANIO_OG	47/129	MiSeq	119.9x	99.5%
3	c.7607G>A (p.Arg2538Gln)	CRANIO_OG	69/133	MiSeq	106.3x	99.3%
4	c.4558del (p.Asp1520Thrfs*11)	RASGENODERM_V3	120/245	MiSeq	197.7x	99.8%
5	c.2395A>T (p.Lys799Ter)	RASGENODERM_V3	150/319	MiSeq	203.1x	99.8x
6	c.1389dup (p.Gly464Argfs*29)	SS_CRANIO_V1	217/419	NextSeq550	279.0x	99.9%
7	c. 7552C>T (p.Gln2518Ter)	SS_CRANIO_V1	100/187	NextSeq550	279.0x	99.8%
8	c.2828_2829del (p.Glu943Valfs*74)	SS_CRANIO_V1	63/131	NextSeq550	89x	96.4%
9	c.6340C>T (p.Gln2114Ter)	Illumina CEX	57/100	HiSeq1500 (2x100)	86.7x	97.3%
10	c.3295_3296del (p.Phe1099Leufs*2)	Exome (CeGaT, Twist Core Exome Plus)	63/124	NovaSeq6000 (2x100)	140x	99.4%
11	c.3771dup (p.Glu1258Argfs*25)	RASGENODERM_V3	120/245	MiSeq	145.1x	99.5%
12	c.1385_1388del (p.Thr462Lysfs*47)	SS_CRANIO_V1	145/190	NextSeq550	175.0x	98.7%
13	c.6053_6057del (p.Pro2018Argfs*12)	RASGENODERM_V3	40/79	MiSeq	147.3x	99.7%
